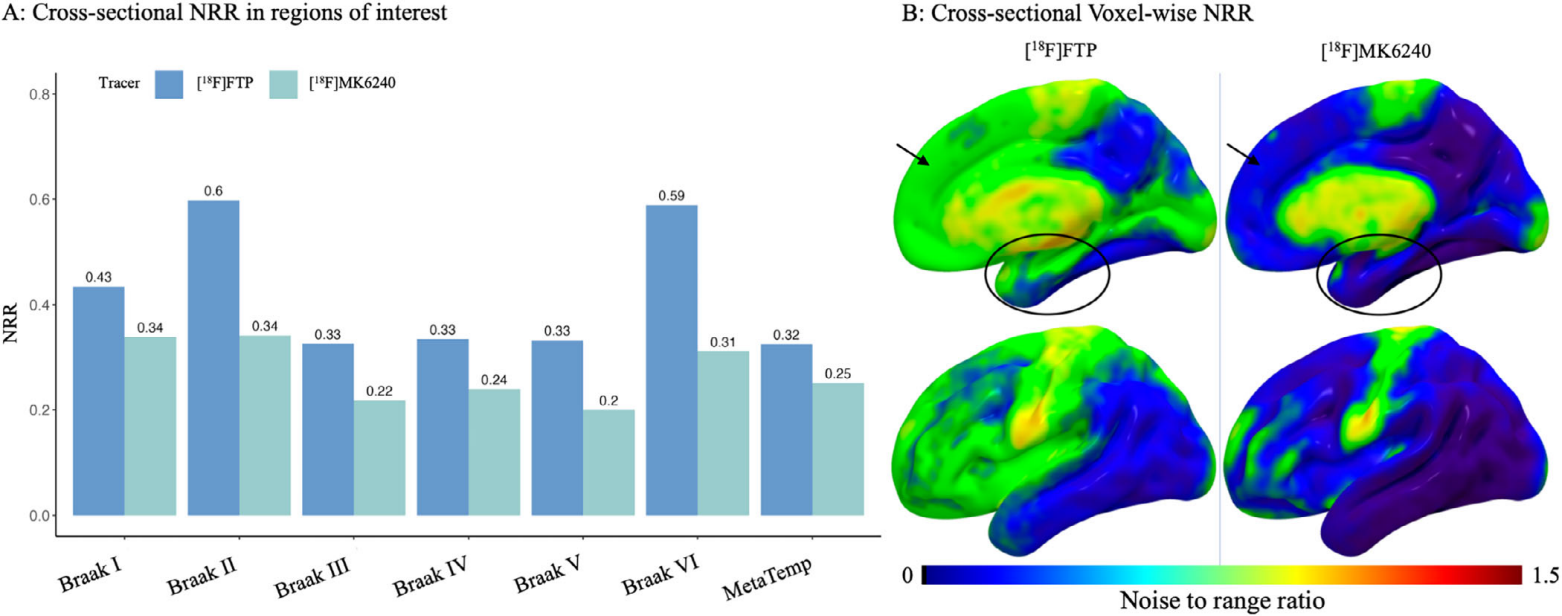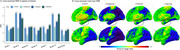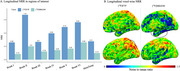# *In vivo* detectability of tau‐PET tracers in Alzheimer's disease

**DOI:** 10.1002/alz70856_106041

**Published:** 2026-01-08

**Authors:** Cécile Tissot, Nesrine Rahmouni, Hsin‐Yeh Tsai, Joseph Therriault, Arthur C. Macedo, Stijn Servaes, Jenna Stevenson, Firoza Z Lussier, Jacob Ziontz, Peiwei Liu, Lydia Trudel, Brian A. Gordon, Belen Pascual, Val J Lowe, David N. Soleimani‐Meigooni, Hwamee Oh, William E Klunk, Pedro Rosa‐Neto, William J. Jagust, Tharick A Pascoal, Suzanne L. Baker

**Affiliations:** ^1^ Lawrence Berkeley National Laboratory, Berkeley, CA, USA; ^2^ McGill University, Montreal, QC, Canada; ^3^ University of Pittsburgh, Pittsburgh, PA, USA; ^4^ University of California, San Francisco, San Francisco, CA, USA; ^5^ University of California, Berkeley, Berkeley, CA, USA; ^6^ Washington University in St. Louis, School of Medicine, St. Louis, MO, USA; ^7^ Houston Methodist Research Institute, Houston, TX, USA; ^8^ Mayo Clinic, Rochester, MN, USA; ^9^ Brown University, Providence, RI, USA

## Abstract

**Background:**

Tau‐PET tracers are essential for visualizing pathology in Alzheimer's (AD). High detectability to tau is crucial for early detection and monitoring of tau deposition. This study compares the noise to dynamic range ratio (NRR) of [^18^F]FTP, [^18^F]MK6240, [^18^F]PI2620, and [^18^F]RO948, cross‐sectionally and longitudinally.

**Methods:**

460 individuals from the HEAD study (23 cognitively unimpaired (CU) young, 249 CU old and 188 cognitively impaired) underwent [^18^F]FTP and [^18^F]MK6240 tau‐PET scans; 94 additionally received [^18^F]PI2620 and [^18^F]RO948. A subset of 28 individuals (15 CU and 13 CI) underwent [^18^F]FTP and [^18^F]MK6240 follow‐up scans (1.5 ± 0.1 years later). Annual change was measured as [(followup‐baseline)/time between scans].

Noise was calculated as the standard deviation (SD) of CU Aβ‐ participants aged ≤65 (SD_CUAβ‐≤65_). The dynamic range was calculated as the SD across all subjects (SD_range_). A lower the NRR (=SD_CUAβ‐≤65_/SD_range_) indicates more detectability. Analyses were performed at both region‐of‐interest and voxel‐wise levels.

**Results:**

Across the whole cohort, [^18^F]MK6240 exhibited lower NRR than [^18^F]FTP in all regions. Voxel‐wise, differences were most pronounced in the frontal medial temporal regions (Figure 1). Within the four‐tracers subset, [^18^F]PI2620 and [^18^F]FTP showed the highest NRR in Braak II, followed by [^18^F]RO948 and [^18^F]MK6240. In Braak IV‐VI, [^18^F]MK6240 consistently demonstrated the lowest values, followed by [^18^F]PI2620, [^18^F]FTP and [^18^F]RO948. Similarly, in metatemporal‐ROI and Braak III, [^18^F]MK6240 remained the lowest, however followed by [^18^F]FTP, [^18^F]PI2620 and [^18^F]RO948 (Figure 2). Lower [^18^F]MK6240 NRR was due to larger SD_range_, while [^18^F]FTP's lower values stemmed from smaller SD_CUAβ‐≤65_. Conversely, high [^18^F]PI2620 and [^18^F]RO948 values are driven by larger SD_CUAβ‐≤65_. Longitudinal analyses further confirmed that [^18^F]MK6240 exhibited the lowest NRR across the entire brain, including AD‐related regions, with voxel‐wise differences mainly in the temporal and parietal lobes.

**Conclusion:**

Tau‐PET tracers exhibit significant variability in detectability. [^18^F]MK6240 consistently demonstrated lower NRR, implying better detectability across all regions, cross‐sectionally and longitudinally. Despite differences in NRR, [^18^F]FTP, [^18^F]RO948 and [^18^F]PI2620 followed similar pattern. These results provide insights into the differential tracer detectability, helping guide their optimal use in detecting and tracking tau pathology. Ongoing follow‐up scans will further clarify longitudinal tracer detectability and its implications for tau progression in AD.